# The Weather-Beaten Dorsal Hand Clinical Rating, Shadow Casting Optical Profilometry, and Skin Capacitance Mapping

**DOI:** 10.1155/2013/913646

**Published:** 2013-10-03

**Authors:** Marie Delvenne, Claudine Piérard-Franchimont, Laurence Seidel, Adelin Albert, Gérald E. Piérard

**Affiliations:** ^1^Laboratory of Skin Bioengineering and Imaging (LABIC), Department of Clinical Sciences, Liège University, 4000 Liège, Belgium; ^2^Department of Medical Informatics and Biostatistics, University of Liège, 4000 Liège, Belgium; ^3^Department of Dermatology, University Hospital St Jacques, 25030 Besançon, France

## Abstract

Laypeople commonly perceive some skin xerosis and withering (roughness) changes during winter on some parts of the body, particularly on the dorsal hands. The aim of the study was to assess the withered skin surface changes occurring during the four seasons. A total of 47 menopausal women completed the study. A group of 31 volunteers were on hormone replacement therapy (HRT) and 16 were out of HRT. Skin xerosis and scaliness were rated clinically. In addition, skin whitening was assessed by computerized shadow casting optical profilometry and by skin capacitance mapping. The volunteers were not using topical creams and over-the-counter products on their hands. Marked changes, recorded over the successive seasons, corresponded to patchy heterogeneous stratum corneum hydration and heterogeneous skin surface roughness changing over seasons; they likely resulted from changes in the environmental temperature and atmosphere moisture. The severity of the changes revealed by clinical inspection was not supported by similar directions of fluctuations in the instrumental assessments. This seemingly contradiction was in fact due to different levels of scale observation. The clinical centimetric scale and the instrumental inframillimetric scale possibly provide distinct aspects of a given biological impact.

## 1. Introduction

Seasonal variations in environmental conditions are prone to alter the skin presentation particularly on the legs, face, and back of the hands [1, 2]. For laypeople, the resulting aspect and feel are described as a dry and rough skin. Various clinical scales have been designed for rating the xerotic harsh conditions [3]. 

The current overwhelming trend steering dermatology aims toward making the descriptions more scientific and clearly identifiable. The skin microrelief and its seasonal withered aspect are conveniently assessed using a series of dedicated noninvasive and scientifically validated methods [4–6]. One of these relies on the collection of negative replicas from the skin surface. The microrelief profile is then conveniently quantified using shadow casting optical profilometry (SCOP). The SCOP procedure for skin analysis consists of lighting the sample by a parallel light source with a defined incident angle. The microrelief generates shadows which are wider when skin peaks and crests are taller [7–9]. The process averages the scannings of a series of parallel lines of 2D assessments.

 Both the relative moisturization of the upper stratum corneum (SC) and the pattern of the skin microrelief are conveniently recorded by skin capacitance mapping/imaging (SCMI). The method was previously described in details [10–17]. In practice, the real-time SCMI nonoptical images are acquired and displayed on a computer screen where capacitance values are presented as pixels in a range of 256 gray levels. When a close contact is secured between the probe and the skin surface, the darker pixels correspond to high capacitance (moisturized) spots, and the clear ones to lower capacitance values. The SCMI-derived mean gray level (MGL) is representative of the average skin surface hydration [12].

 The purpose of this study was to rate clinically and to assess objectively some variations in the skin surface landmarks on the dorsal hands of menopausal women over a 11-month period. The women received or not hormone replacement therapy (HRT). The heterogeneity in SC hydration and the skin microrelief were assessed by combining SCMI image analysis and the SCOP surface-shadowing.

## 2. Material and Methods

### 2.1. Design

The present observational study was approved by the Ethic Committee of the University Hospital of Liège, and the procedure was performed in accordance with the Declaration of Helsinki. The clinical and noninvasive instrumental procedures were conducted with the understanding and consent of all volunteers. 

A total of 60 women with predominant outdoor occupational activities in open-air markets were enrolled in the study. For various reasons, not all subjects attended every assessment, and 13 of them did not completed the full program of the study. Among the 47 women who completed the study, 31 (66%) volunteers aged 53 ± 2 years received HRT. The other group of 16 volunteers aged 51 ± 3 years were out of HRT. The drop-out subjects were 5 women on HRT and 8 out of HRT. None of the women were using anytime hand creams and over-the-counter topical medications. There was no oral supplementation and a washout period was not necessary before starting the study. They were not usually wearing gloves. Four quarterly clinical and noninvasive instrumental assessments were performed starting in June-July. These evaluations corresponded to the summer, fall, winter, and spring periods, respectively. In each subject, the mid part of the back of the dominant hand was assessed clinically using both SCMI and SCOP. The participants of the HRT and non-HRT groups were blinded for the assessments at each seasonal collection.

 On attendance for assessment, each subject first had to remain relaxed for 20–35 min in the Laboratory of Skin Bioengineering and Imaging under controlled temperature (20 ± 1°C) and humidity (55 ± 2%). In a first assessment step, a SkinChip (L'Oréal, Paris, France) probe was applied to the skin for 5 s at the most. The SCMI determination was obtained by the SkinChip device providing images of skin capacitance measurements every 50 *μ*m. 

**Figure 1:**
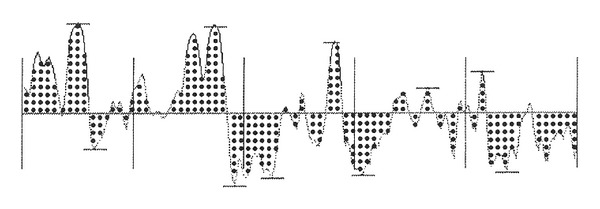
Profilometric parameters of skin microrelief. Ra is the dotted area, RN is the number of peaks above 0.1 mm, and Rz is the mean depth of the roughness profile (difference between the highest peak and deeper furrow in each of the 5 sectors).

In a second assessment step, a negative silicon replica (Silflo resin, Flexico Development Lim, Herts, UK) was collected as previously described [18, 19]. Each sample was illuminated by a floodlight (Highlight 3000 Olympus, Omnilabo, Brussels, Belgium) oriented at a 38° incidence angle. The lighting generated shadows, the width of which reflected the height of peaks and crests of the skin withering. A computerized image analyzer (Dermatec, Paris, France), working with gray-level discrimination, recorded surface topography parameters (Figure 1) Ra represented the mean roughness value, that is, the area above and below an average line through the center of the profile; Rz was the mean depth of roughness, that is, the average difference between the minimum and maximum heights in each of 5 adjacent sectors; Rn corresponded to the number of peaks or crests greater than 100 *μ*m.

Following the biometeorological evaluations, a visual assessment was further performed. Xerotic and flaking skin was quite easily visualized after removing skin surface lipids. This was achieved following a swabbing method using cotton wool with propan-2-ol and allowing the excess alcohol on the skin surface to evaporate during 4 minutes before visual assessment [3]. 

The same trained assessor performed each assessment in order to guarantee continuity of scoring. The reproducibility of these records was periodically checked by comparing these scores with those given by a second expert assessor. Flaking xerotic skin was assessed using a predetermined scale (Table 1). This scale helped visual assessments of a series of parameters by examining three distinct visual aspects of flaking skin independently: (a) the grade of skin flakes, (b) their density, and (c) the area covered by the flakes. The clinical flaking score (CFS) was calculated by adding scores for each of the three aspects [3]. 

 Data were expressed as means and standard deviations (SD). The minimum and maximum values were recorded as well as the medians. The Student's *t*-test and the Kruskal-Wallis test were used for comparing the two volunteer groups in the time-related observations. The relationships between the recorded parameters at each evaluation time were assessed using both the Pearson and the nonparametric Spearman correlation coefficients. The seasonal effect for each group of volunteers was assessed using the two-way analysis of variance (ANOVA-2). Multiple comparisons were performed according to the Scheffe test. The initial summer assessments in June-July served as references. Results were considered significant at the 5% critical level (*P* < 0.05). Calculations were performed using the SAS version 9.3 software (SAS Institute, Cary, NC, USA). 

## 3. Results

**Figure 2:**
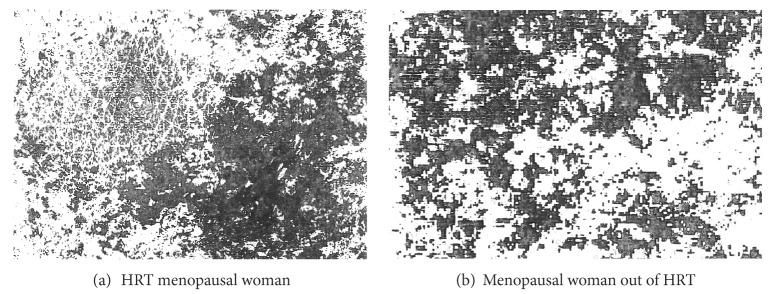
Examples of heterogeneous patterns of skin capacitance mapping on dorsal hands in winter.

Data about Ra, Rn, Rz, and SCMI-MGL are presented in Tables 2, 3, 4, and 5. The SCMI showed heterogeneous patterns of pixel darkness, particularly at the winter assessment in about 25–35% of women irrespective of their HRT status (Figure 2). For all instrumental assessments a periodic seasonal change during this study was clearly evidenced in most volunteers.

### 3.1. Seasonal Effect

The average CFS fluctuated over the seasons. In menopausal women out of HRT, it reached 3.1 ± 0.7 in summer, 3.9 ± 1.3 at fall, 7.4 ± 1.2 in winter, and 4.4 ± 2.3 in spring. In HRT recipients, the CFS averaged 3.3 ± 0.5 in summer, 3.5 ± 0.6 at fall, 6.3 ± 0.8 in winter, and 4.0 ± 1.6 in spring.

SCMI-MGL showed seasonal fluctuations in women receiving HRT (*P* = 0.0014) or not (*P* = 0.0002). Ra showed no seasonal variations in HRT women. By contrast, a prominent effect (*P* < 0.0001) was yielded on Ra in women out of HRT. Rn showed no seasonal effect in HRT women. By contrast, a major effect (*P* < 0.001) was observed in women out of HRT. Rz showed seasonal variations in women receiving HRT (*P* = 0.047) or not (*P* < 0.0001).

 When considering both groups of women, SCMI-MGL showed a seasonal effect (*P* < 0.0001) without any group difference. The evaluations of each roughness parameter were different in each women group for Ra (*P* < 0.001), Rn (*P* < 0.001), and Rz (*P* = 0.013).

### 3.2. Intergroup Comparisons at Each Season

No significant differences were yielded between the CFS of the two groups of women anytime during the study. By contrast, some significant intergroup differences were present in the biometeorological assessments.

During summer, no intergroup differences were yielded for Rz and SCMI-MGL. By contrast, both Ra and Rn values were significantly higher (*P* = 0.012 and 0.0071, resp.) in the HRT recipients.

 At fall, significant differences were present in each of the four parameters between the two groups of women. The SCMI-MGL was higher (*P* = 0.0021) in women out of HRT. By contrast, the roughness parameters had higher values in the HRT group (Ra, *P* = 0.0024; Rn, *P* = 0.0082; Rz, *P* = 0.0002).

 In winter, no difference was observed between the two groups regarding Rz and SCMI-MGL. By contrast, both Ra (*P* = 0.024) and Rn (*P* = 0.012) were higher in the nonsupplemented women.

 In spring, no significant differences were yielded for Ra and Rz between the two groups. Values of Rn (*P* = 0.036) and SCMI-MGL (*P* = 0.012) were higher in women out of HRT.

### 3.3. Correlations between Skin Capacitance and Roughness Parameters in both Groups at Each Season

During summer, HRT women showing high SCMI-MGL were significantly associated with low roughness (Ra, *P* = 0.0392; Rn, *P* < 0.001; Rz, *P* < 0.0001). In women out of HRT, a high SCM-IMGL was associated with low values of Ra (*P* = 0.0013) and Rz (*P* < 0.0001).

 At fall, HRT women exhibiting high SCMI-MGL exhibited low values of Ra (*P* = 0.0004), Rn (*P* < 0.0001), and Rz (*P* < 0.0001). In women out of HRT, a positive correlation was found between Ra and Rn (*P* = 0.029) and between Rn and Rz (*P* = 0.007).

 In winter, HRT women with high SCMI-MGL had low values of Ra (*P* < 0.0001), Rn (*P* < 0.0001), and Rz (*P* < 0.0001). The women out of HRT showing high SCMI-MGL had a low Rz value (*P* = 0.0016).

 In spring, HRT women with high HCMI-MGL had low values of Rz (*r* < 0.001). No correlations were found between SCMI-MGL and any roughness parameter in women out of RHT. 

## 4. Discussion

The normal SC binds water for ensuring a soft and smooth surface. It corresponds to a sturdy tissue of tightly packed coherent corneocytes. Its components have the capacity to bind water and to prevent water evaporation from the skin surface. The upshot of previous intense investigations was the perception that the dead, anucleated corneocytes remained indeed active metabolically. As corneocytes move to the skin surface, a remarkable series of structural and enzymatic changes take place [1, 2]. These events are tightly regulated and ensure homeostasis facing the hostile physical and chemical environment. Some abnormalities in such SC processing produce a xerotic and rough skin surface potentially leading to fine cracking and fissuring.

Many intrinsic factors influence the appearance and function of the skin surface. Similarly, many extrinsic environmental factors exert a physiological effect either directly or indirectly. For instance, xerotic conditions occur more commonly during winter season. The direction of the environmental temperature variations is opposite to the change in global flaking. Many studies attempted to attribute skin dryness and chapping to further environmental factors including relative humidity and absolute humidity. Atmospheric moisture and dew point are thus expected to exert some influences on the skin condition [3, 20, 21]. 

It is a common place for laypeople to complain from “dry skin” during cold seasons. Obviously, the geographic environment and meteorology contribute to the skin ailment. Few studies have been performed in the past for objectivating the skin surface impact of the successive seasons. The present study was undertaken on specific groups of early menopausal women receiving or not HRT. The HRT supplementation indeed exerts some effects on the skin [22, 23]. The objective methods were the quantifications of the SC hydration (skin capacitance) and skin withering/roughness/harshness using clinical ratings as well as determinant of skin capacitance and the Ra, Rn, and Rz parameters on skin replicas. All these procedures were noninvasive. In most seasons and in both women groups, the roughness parameters Ra, Rn, and Rz appeared correlated in the present study. A high capacitance level was commonly associated with a discrete roughness in HRT recipients at any season. Such correlations were less obvious in women out of HRT.

Clinical ratings from subjective assessment scales should never use any more than a five-point descriptor scale, because assessor proficiency steadily decreases as the number of assessment classes increases above this level. The composite scale presently used enabled a representative assessment of the skin condition [3]. The clinical rating globally yielded seasonal variations consistent with the volunteer perceptions of a xerotic aspect of the dorsal hands during successive seasons. Contrary to common expectations, the instrumental assessment were at variance, particularly in the distinction of skin reactivity according to the intake or avoidance of HRT. 

 The present study clearly distinguishes a different reactivity of the SC of menopausal women to environmental changes according to the intake or not of HRT. Seasonal variations appeared less impressive in HRT recipients. Surprisingly, during summertime, both Ra and Rn were more pronounced in the HRT women than in nonsupplemented women. No difference was disclosed with women out of HRT as far as the mean skin capacitance and Rz were concerned. At fall, all four biometeorological parameters were affected differently in both groups of women. In particular, skin capacitance was higher in women out of HRT. During wintertime, both Ra and Rn became higher in women out of HRT. During that period, no intergroup difference was evidenced for both mean capacitance and Rz. In spring, both skin capacitance and Rn were higher in women out of RHT while no intergroup group differences were yielded for Ra and Rz. In sum, mean skin capacitance showed little differences between both groups of menopausal women. By contrast, parameters of skin roughness showed larger intergroup differences, and variations were in opposite directions during colder and warmer seasons.

 In short, in the group of menopausal women out of HRT, the seasonal effect combined lower capacitance and increased roughness as shown by Ra, Rn, and Rz during winter. In HRT recipients, a similar chronobiology was evidenced for both skin capacitance and Rz. Hence, HRT appeared to abate some manifestations of seasonal dry skin on the dorsal aspect of the hands. In this study, the effects of the external environment were specifically considered. This study demonstrates seasonal changes in the prevalence of a xerotic flaking skin condition, and the incidence appears to be governed by environmental temperature and atmospheric moisture content. However, the indoor environment possibly contributed to the overall skin condition. Indeed, alternate exposures to both normalized indoor and variable natural outdoor environments undoubtedly put the skin under stress [3]. They probably affect its general condition, particularly under extreme temperatures and humidity. The xerotic flaking skin condition possibly results in part from such cyclic exposures. 

## Figures and Tables

**Table 1:** Subjective scoring scale of flaking skin*.

Flake size	
0: Dulled powdery appearance, no flakes visible	
1: Very small but visible flakes	
2: Intermediate sized flakes (at least two times size of I)	
3: Large flakes with obvious curling edges	
Flake density	
0: No flakes, powdery only	
1: Sparsely distributed flakes (size I to 3)	
2: Nonuniform covering of flakes	
3: Flakes in close proximity, with uniform covering of flaking area	
Area of cover	
0: No flaking	
1: Less than one-third of swabbed area flaking	
2: Once- to- two-thirds of swabbed area flaking	
3: Over two-thirds of swabbed area flaking	

*Flaking score = sum of scores of flake size, density, and area of cover. Possible scores: 0, 3, 4, 5, 6, 7, 8, 9.

**Table 2:** Ra values.

Season	*N*	Mean	SD	Min	Median	Max
HRT						
Summer	31	9.57	4.45	2.62	9.46	19.27
Fall	31	8.60	3.71	2.38	8.09	16.58
Winter	31	8.89	4.24	1.99	8.59	16.95
Spring	31	9.12	3.73	2.24	8.48	15.59

Non-HRT						
Summer	16	6.35	2.88	1.98	6.06	12.13
Fall	16	5.37	2.12	1.73	5.37	8.38
Winter	16	12.18	5.14	8.10	10.14	28.42
Spring	16	10.82	2.64	6.33	10.05	16.27

**Table 3:** Rn values.

Season	*N*	Mean	SD	Min	Median	Max
HRT						
Summer	31	3.71	4.02	0.00	2.00	13.00
Fall	31	5.74	5.28	0.00	4.00	17.00
Winter	31	5.77	4.67	0.00	6.00	14.00
Spring	31	5.26	4.65	0.00	3.00	13.00

Non-HRT						
Summer	16	0.81	1.05	0.00	1.00	4.00
Fall	16	2.00	1.51	0.00	2.00	5.00
Winter	16	9.38	4.06	0.00	10.50	14.00
Spring	16	9.31	3.42	3.00	9.00	15.00

**Table 4:** Rz values.

Season	*N*	Mean	SD	Min	Median	Max
HRT						
Summer	31	18.63	10.36	5.80	17.80	46.60
Fall	31	23.37	14.08	9.40	17.80	59.50
Winter	31	27.09	17.70	5.80	18.40	63.20
Spring	31	23.05	15.00	5.80	18.00	55.30

Non-HRT						
Summer	16	13.23	3.94	7.80	13.00	21.40
Fall	16	11.36	3.46	6.80	10.60	17.00
Winter	16	28.40	14.28	4.02	30.25	58.40
Spring	16	24.39	11.73	8.40	21.75	47.60

**Table 5:** Non-optical capacitance imaging (mean gray level).

Season	*N*	Mean	SD	Min	Median	Max
HRT						
Summer	31	122.61	36.96	64.00	129.00	186.00
Fall	31	109.29	34.95	57.00	115.00	176.00
Winter	31	92.35	37.40	29.00	101.00	153.00
Spring	31	104.19	36.75	50.00	96.00	163.00

Non-HRT						
Summer	16	127.94	25.01	74.00	130.00	170.00
Fall	16	138.75	11.46	122.00	138.00	161.00
Winter	16	105.94	27.38	68.00	106.50	150.00
Spring	16	129.19	13.61	109.00	130.50	157.00
